# Modelling glioblastoma tumour-host cell interactions using adult brain organotypic slice co-culture

**DOI:** 10.1242/dmm.031435

**Published:** 2018-02-01

**Authors:** Maria Angeles Marques-Torrejon, Ester Gangoso, Steven M. Pollard

**Affiliations:** MRC Centre for Regenerative Medicine and Edinburgh Cancer Research UK Cancer Centre, University of Edinburgh, 5 Little France Drive, Edinburgh, EH16 4UU, UK

**Keywords:** Glioblastoma, Adult brain, Niche, Slice culture, Quiescence, Proliferation

## Abstract

Glioblastoma multiforme (GBM) is an aggressive incurable brain cancer. The cells that fuel the growth of tumours resemble neural stem cells found in the developing and adult mammalian forebrain. These are referred to as glioma stem cells (GSCs). Similar to neural stem cells, GSCs exhibit a variety of phenotypic states: dormant, quiescent, proliferative and differentiating. How environmental cues within the brain influence these distinct states is not well understood. Laboratory models of GBM can be generated using either genetically engineered mouse models, or via intracranial transplantation of cultured tumour initiating cells (mouse or human). Unfortunately, these approaches are expensive, time-consuming, low-throughput and ill-suited for monitoring live cell behaviours. Here, we explored whole adult brain coronal organotypic slices as an alternative model. Mouse adult brain slices remain viable in a serum-free basal medium for several weeks. GSCs can be easily microinjected into specific anatomical sites *ex vivo*, and we demonstrate distinct responses of engrafted GSCs to diverse microenvironments in the brain tissue. Within the subependymal zone – one of the adult neural stem cell niches – injected tumour cells could effectively engraft and respond to endothelial niche signals. Tumour-transplanted slices were treated with the antimitotic drug temozolomide as proof of principle of the utility in modelling responses to existing treatments. Engraftment of mouse or human GSCs onto whole brain coronal organotypic brain slices therefore provides a simplified, yet flexible, experimental model. This will help to increase the precision and throughput of modelling GSC-host brain interactions and complements ongoing *in vivo* studies.

This article has an associated First Person interview with the first author of the paper.

## Abstract (precis)

**Editor's choice:** A new *ex vivo* experimental approach to explore how glioblastoma stem cells interact with distinct brain microenvironments, with potential use a as preclinical model using new anticancer agents.

## INTRODUCTION

Glioblastoma multiforme (GBM) is a highly aggressive malignant brain tumour. It is the most malignant form of glioma. Standard treatments involve combined surgery, radiotherapy and adjuvant temozolomide (TMZ) chemotherapy (Stupp et al., 2005), but survival rates are extremely poor. Various obstacles hamper development of effective therapies, including pervasive tumour cell infiltration, genetic heterogeneity (both intra- and intertumoural), therapeutic resistance, blood-brain barrier and lack of biological understanding of the disease. Improved experimental models will help address some of these issues.

GBM tumour cells disseminate widely across many brain regions, often following neuronal tracts and vasculature. Cells are therefore exposed to diverse microenvironments, such as specific repertoires of cell matrix and growth factors, or cellular niches (e.g. perivascular, invasive or hypoxic). These environmental cues steer glioma stem cell (GSC) fate, affecting quiescence, proliferation, survival and differentiation pathways (Codrici et al., 2016; Gilbertson and Rich, 2007; Okawa et al., 2016). Modelling these various tumour cell-host brain interactions is therefore vital for improved understanding of disease biology and development of new therapeutic strategies.

GSCs highjack many molecular programmes that regulate neural stem cell self-renewal. Improved understanding of mechanisms controlling neural stem cell fate will thus likely lead to new insights into the disease and identification of crucial therapeutic targets. Neural stem cells (NSCs) are located within the lateral walls of the forebrain ventricles in a region known as the subependymal zone (SEZ) (Doetsch et al., 1999). The SEZ provides a specific niche that sustains the NSCs throughout life. NSCs are exposed to a myriad of cell-cell signals and extracellular matrix (ECM) interactions that steer NSC fate, such as endothelial cells, ependymal cells and cerebral spinal fluid (Mirzadeh et al., 2008; Shen et al., 2008; Silva-Vargas et al., 2016). Understanding how tumour cells respond to a normal SEZ is important, as this might be a region harbouring a reservoir of tumour cells (Piccirillo et al., 2015).

Patient-derived GSCs can be routinely expanded *in vitro* using culture media developed for NSCs, either in suspension or adherent culture (Galli et al., 2004; Hemmati et al., 2003; Lee et al., 2006; Pollard et al., 2009; Xie et al., 2015). Orthotopic transplantation of freshly isolated or cultured GSCs into the adult rodent brain using stereotaxic surgery is the ‘gold standard’ method to test tumour-initiating potential. However, animal surgery and transplantation deep into the brain provides limited scope to track live-cell behaviours. Typically, these experiments take weeks or months and are nontrivial to set up. They do not enable direct inspection of single cell behaviours, such as invasion, monitoring of quiescence and differentiation, or responses to genetic or chemical perturbations. These practical constraints have limited the scale and scope of studies aimed at understanding and treating gliomas. To address this, we explored the utility of organotypic slice cultures to monitor GSC-host interactions.

Organotypic brain slice cultures were first developed in the 1960s (Crain, 1966). Since then, they have been widely used by neuroscientists, particularly in studies of neuronal function and circuits (reviewed in Humpel, 2015). Microdissected regions are cultured above a semipermeable membrane in a cell culture insert and exposed to serum containing medium from below. An example of their successful use are studies using hippocampal slices cultures; this technique is widely deployed for studies of synaptic plasticity and memory (Gähwiler et al., 1997). Organotypic slice cultures overcome some of the difficulties of *in vivo* studies as they provide *ex vivo* access to brain tissue architecture, while still enabling direct observation and cell manipulations in the culture dish (Humpel, 2015). Slice cultures have also been used to explore the response of glioma cells to brain tissue, particularly to explore mechanisms of infiltration and migration. However, these have mainly used postnatal brain slices grown in serum or from mice harbouring pre-existing tumours (Minami et al., 2017; Matsumura et al., 2000; Jensen et al., 2016; Ohnishi et al., 1998).

Here, we report improved conditions enabling serum-free culture of adult coronal whole-brain slices in a manner that enables tracking of GSC behaviours over several weeks. Our experimental approach provides a useful new strategy to explore GBM. This model bridges the ‘experimental gap’ between *in vitro* cell culture models and *in vivo* orthotopic transplantations. As an exemplar of the utility of this approach, we confirm engraftment of GSCs around blood vessels in the slice culture and demonstrate how it can be used in preclinical studies of anticancer agents.

## RESULTS

### Whole adult brain coronal slice cultures are viable for weeks in serum-free NSC medium

Most studies employing organotypic slice cultures use postnatal mice and dissect specific regions of the brain (e.g. hippocampus). However, GBM is predominantly a disease of adults and cells disseminate widely across all brain regions. We therefore focused on whole brain slices, reasoning that even short-term viability, for days or weeks, could provide a useful model for testing tumour cell-host brain interactions.

**Fig. 1.**
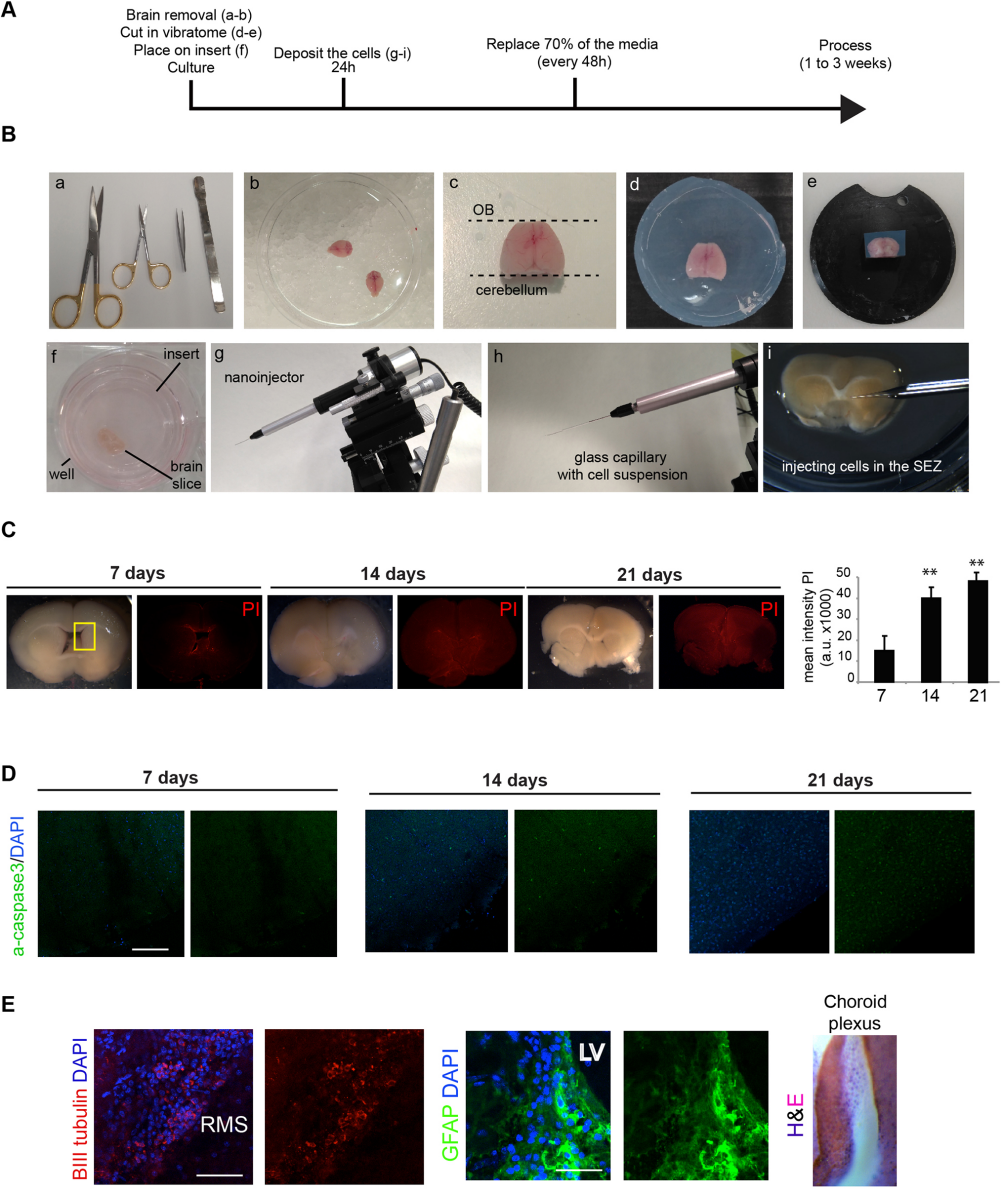
**Overall experimental strategy and tissue processing.** (A) Summary of the experimental procedure to generate slices. (B) Experimental steps in the harvesting, mounting, slicing and injection of brain tissue; (a) scissors, forceps and a spatula were used to isolate and dissect the whole brain; (b) whole adult mice brain on ice following harvesting; (c) dorsal image of a whole brain following removal of the olfactory bulb (OB) and cerebellum; (d) embedded brain in low melting agarose; (e) brain attached to the support of the vibratome; (f) ∼250 µm coronal brain slice placed onto a cell culture insert in a six-well plate with NSC basal medium; (g) nanoinjector mounted on a micromanipulator used for injection of small volumes of cells; (h) mounted glass capillary containing the cell suspension; (i) microinjection of cells into the SEZ of a coronal brain slice on the cell culture insert. (C,D) After 7, 14 and 21 days, tissue was stained for PI (C) and active caspase 3 (D). The boxed area in C shows the SEZ. Quantification of the mean intensity of PI in the brain slices up to 21 days (rightmost in C). (E) Immunocytochemistry following 7 days in slice co-culture for BIII tubulin neuroblasts (red, left), GFAP-positive gliotubes (green, middle), and choroid plexus (H&E, right). Nuclear counterstaining was performed with DAPI in each (blue). CC, corpus callosum; LV, lateral ventricle; RMS, rostral migratory stream; Sep, Septum; SEZ, subependymal zone. Scale bars: 200 μm in D; 100 µm (left) and 10 µm (right) in E. *n*=3, Student’s *t*-test, **P<0.01.

Adult brains were harvested from young adult mice (∼4 weeks) and the olfactory bulbs and cerebellum were removed (Fig. 1A,B). We generated whole-brain coronal sections using a vibratome to cut ∼200 µm slices at the level of the forebrain ventricle (six slices per brain). Each section was placed onto a semipermeable membrane culture insert and cultured in a six-well cell culture plate (Fig. 1B).

Established organotypic brain slice protocols require high levels of serum or growth factors. However, serum exposure will trigger astrocyte differentiation of NSCs (Conti et al., 2005) (Fig. S1A,B). Culture medium lacking serum or exogenous growth factors was therefore tested. This removes the risk of cell fate being primarily directed by additives in the culture medium, rather than endogenous tissue-derived signals. Dying cells were identified at edges of the dissected region by propidium iodide (PI) staining (Fig. 1C) and active caspase 3 immunostaining (Fig. 1D). Serum-free NSC-permissive culture medium could indeed support slices over several weeks (Fig. 1C). By contrast, in basal medium with no N2 or B27 hormonal supplements, the tissue became necrotic within days (Fig. S1C).

Within the healthy coronal sections, we were able to detect BIII tubulin-expressing neuroblasts, gliotubes and choroid plexus, suggesting the tissue retained key features of the neurogenic niche (Fig. 1E). In summary, whole brain coronal slices are viable for several weeks in serum-free medium – much longer than we anticipated – thereby providing an opportunity to assess responses of transplanted GSCs.

### Patient-derived GSCs engraft into the mouse SEZ and retain expression of quiescent NSC markers CD133 and CD9

**Fig. 2.**
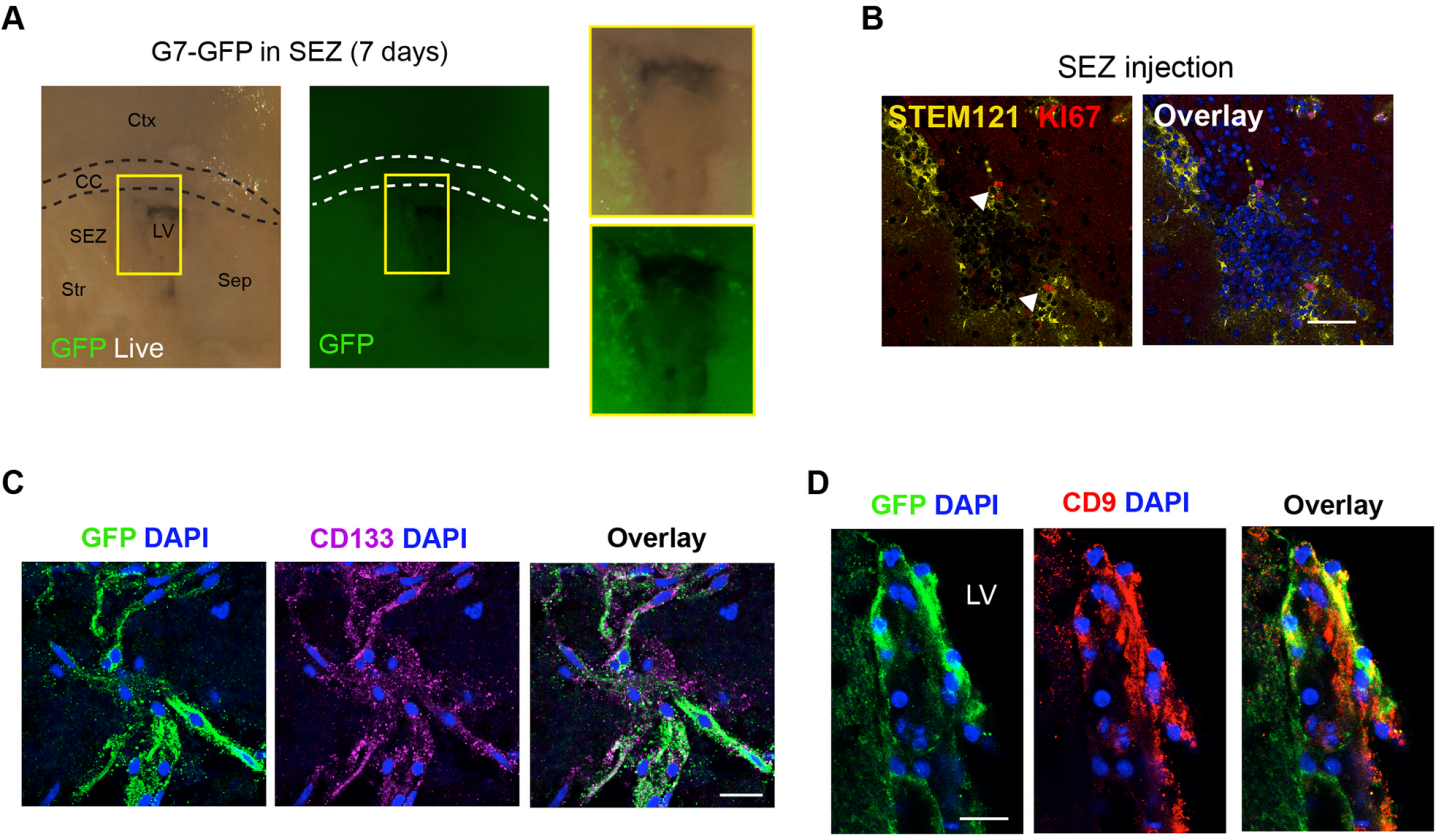
**Human patient-derived GSCs engraft in adult mouse SEZ.** (A) Direct microinjection of human line into adult SEZ and visualization of engrafted live cells using a constitutive GFP reporter. The boxed areas are shown at higher magnification on the right. (B) Immunostaining for human-specific cytoplasmic antigen (STEM121; yellow) and KI67 (red) after 14 days. Arrowheads indicate cells immunopositive for STEM121 and KI67. (C,D), Marker analysis after 21 days of *ex vivo* culture: immunocytochemistry for CD133 (purple) (C) and CD9 (red) (D). Nuclear counterstaining was performed with DAPI (blue). Scale bars: 50 µm in B; 20 µm in C and D.

We next tested the potential of patient-derived human GSCs to engraft into the slices. G7-GFP cells have previously been shown to be highly invasive when transplanted into the striatum of immunocompromised mice (Stricker et al., 2013). We tested different NSC markers *in vitro* before depositing the cells: KI67 (MKI67), OLIG2, SOX2, NES, CD9, and CD133 (PROM1) (Fig. S2A). We first tested microinjection of 10,000 cells into the SEZ (Fig. 2A). One week later, using live cell imaging, we noted large numbers of healthy GFP-expressing cells successfully engrafted. After 2 weeks, slices were fixed and immunocytochemistry (ICC) confirmed that cells were viable and ∼10% were actively proliferating, based on KI67 and STEM121 expression (Fig. 2B). We next assessed the known cancer stem cell marker CD133 in the GFP cells after 3 weeks. Interestingly, CD133-expressing cells were identified in a subset of cells, suggesting that GSCs generate phenotypic heterogeneity in the slices (Fig. 2C).

New molecular markers associated with quiescent NSCs (qNSCs) have recently been uncovered using single cell RNA-seq approaches (Llorens-Bobadilla et al., 2015; Shin et al., 2015). The transmembrane glycoprotein tetraspin (CD9) was identified as a putative marker of qNSCs. The G7 cells within the SEZ in slices expressed CD9 (Fig. 2D) after 21 days. We conclude that human GSCs can engraft, survive and proliferate in whole-brain slice co-cultures for at least 3 weeks, while retaining key cancer stem cell markers.

### Patient-derived GSCs have distinct responses to region-specific adult brain microenvironments

**Fig. 3.**
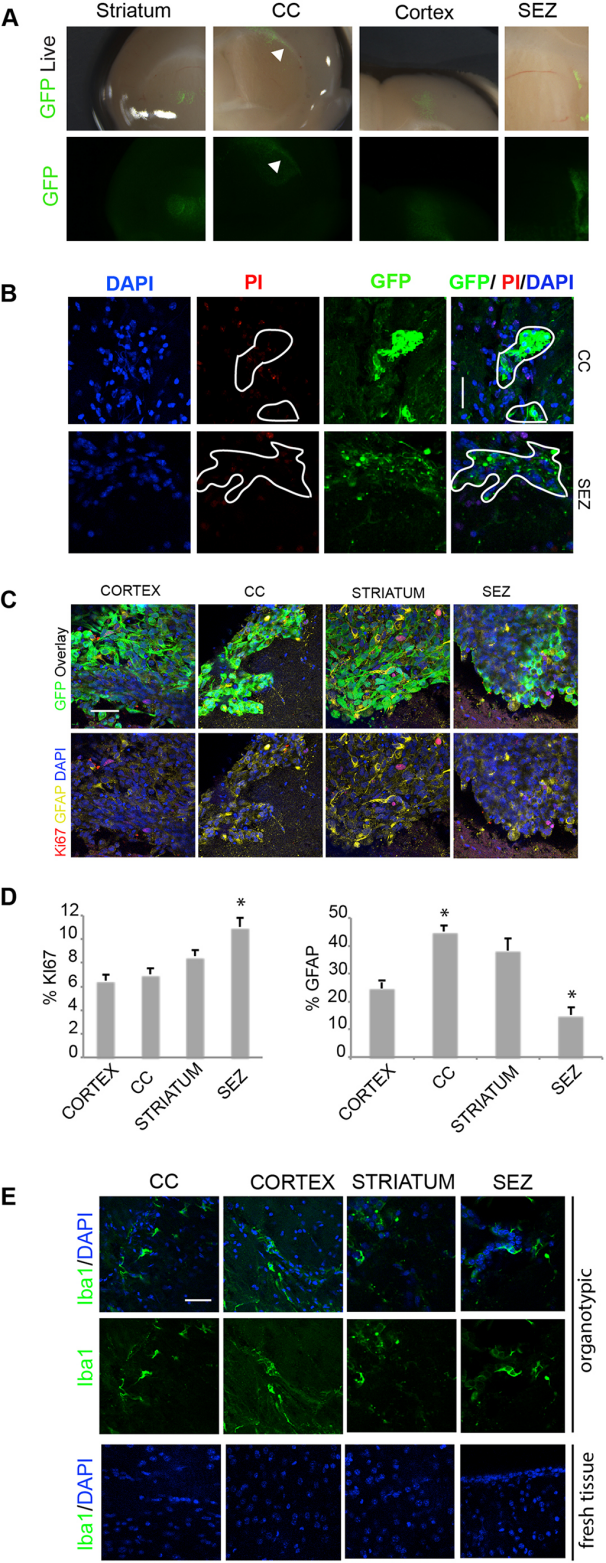
**Differential proliferative responses to human GSCs engrafted into different brain regions.** (A) Live images of G7-GFP human GSCs deposited into distinct regions of the same coronal brain slice after 7 days. Arrowheads indicate cells moving through the CC. (B) Staining for PI to test necrotic tissue surrounding GSCs in tissue in the CC and SEZ. (C) Immunocytochemistry for GFAP (yellow), KI67 (red) and GFP (green). (D) Quantification of the percentage of proliferating and differentiating cells (KI67 and GFAP, respectively). (E) Immunocytochemistry for IBA1 (green) in the CC, cortex, striatum and SEZ. Nuclear counterstaining was performed with DAPI (blue). Scale bars: 50 µm. *n*=3, Student’s *t*-test, *P<0.05.

We next compared how cells would respond in the SEZ versus other brain regions in terms of their proliferation and differentiation. Four distinct regions were tested: the striatum, corpus callosum (CC), cortex and SEZ (Fig. 3A). One week after microinjection into a lateral region of the CC, we noted many G7 cells aligning with and dispersing across the white matter tracts and displaying infiltrative morphology. PI staining of technical replicates was used to assess the viability of CC and SEZ adjacent regions, and we did not observe any significant cell death in these regions (Fig. 3B). Transplanted cells displayed reduced KI67 and increased GFAP compared to cells deposited in parallel within the SEZ of the same slice (Fig. 3C,D). Similar results were observed for cells within the cortex and striatum. Thus, slice cultures provide a convenient method to quickly assess responses of GSCs to the diverse anatomical microenvironments within the adult brain. This enables future exploration of various signals regulating cell fate within the SEZ niche.

Microglia in the adult brain are often activated during injury. In order to check whether there was pervasive, and/or region-specific activation of microglia in the slices, we assessed the activated microglia marker ionized calcium binding adaptor molecule 1 (IBA1; AIF1) using immunohistochemistry (Fig. 3E). As expected, we noted activation; however, this was general at the surface of the slices and was not enriched in any specific region.

### Mouse glioblastoma-initiating cells engraft into the SEZ and can juxtapose to endothelial cells

**Fig. 4.**
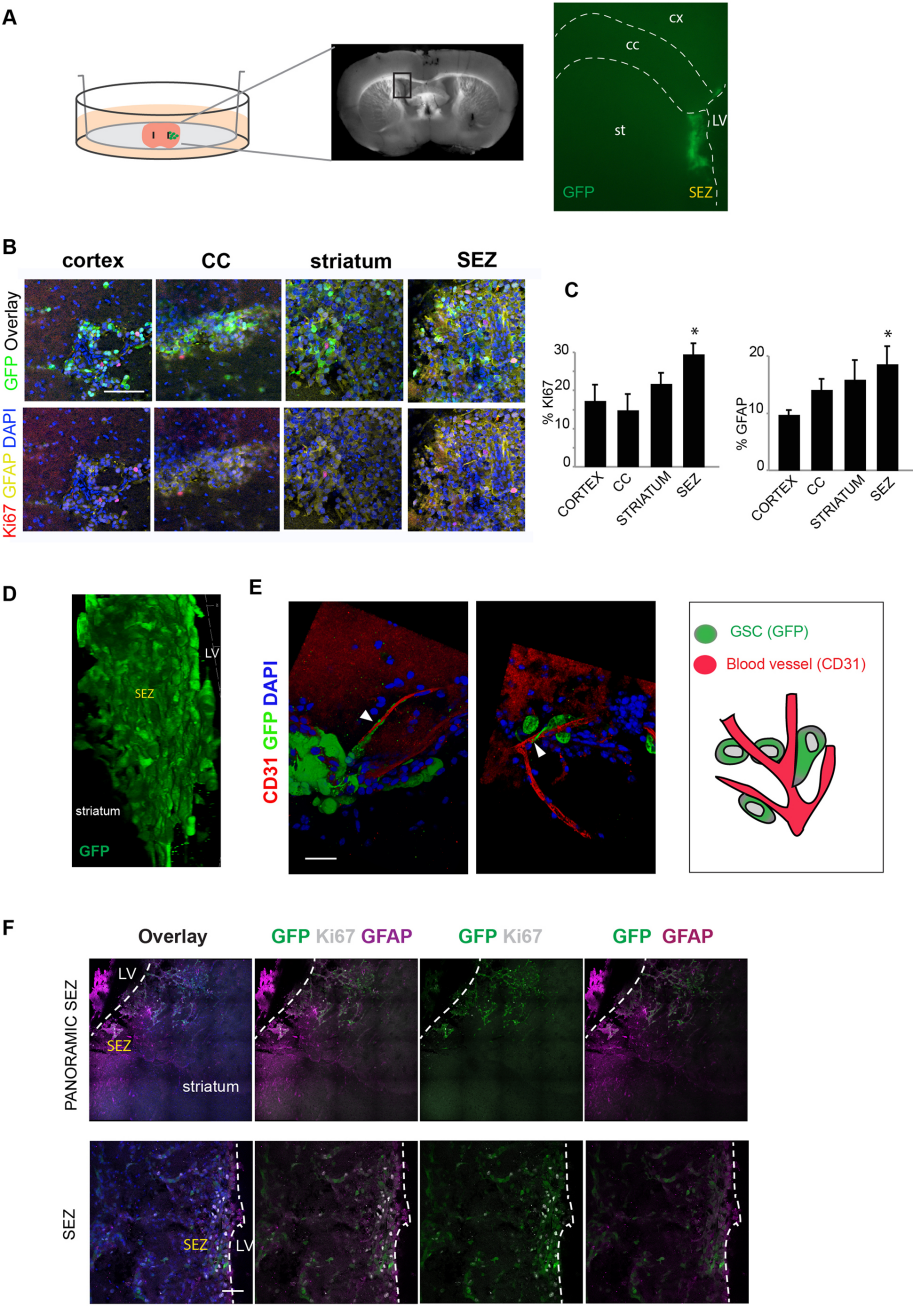
**GSCs in their niche.** (A) Schematic of the experimental setup: co-culture of GSCs and a whole brain coronal section. The boxed area in the section is shown enlarged on the right, indicating the panoramic position of the GSCs (GFP, green) in the SEZ after deposition. (B) Immunostaining for GFAP (yellow), Ki67 (red) and GFP (green) in the cells deposited in different parts of the brain slices. Nuclear counterstaining was performed with DAPI (blue). (C) Quantification of the percentage of proliferating and differentiating cells (Ki67 and GFAP, respectively). (D) 3D image showing the engraftment and infiltration of the IENS-GFP in the brain slice after 5 days. (E) 3D image with immunostaining of the IENS-GFP contacting the blood vessels: GFP (green), CD31 (red) and nuclear counterstaining with DAPI (blue). Arrowheads indicate contact of GSCs with blood vessels. Schematic of the interaction of GSCs with brain blood vessels (right). (F) Top: panoramic view of IENS-GFP cells in SEZ with immunostaining for GFP (green), Ki67 (white), GFAP (purple) and nuclear counterstaining with DAPI (blue). Bottom: detail from the images in the top row. Cells are proliferating and expressing GFP, the astrocytic marker GFAP and the proliferative marker Ki67. LV, lateral ventricle. Scale bars: 100 µm in B; 20 µm in E; 50 µm in F. *n*=3, Student’s *t*-test, *P<0.05.

To minimize disruption of the niche and to enable injection of smaller volumes/numbers of cells (∼100 cells in ∼40nl), we performed transplantation of cells using a microinjection pump connected to a pulled glass capillary (Fig. 1A,B). This enabled more precise and localized injection into the walls of the lateral ventricle (Fig. 4A). We used a previously characterized mouse tumour-initiating cell line, termed IENS-GFP (Ink4A/Arf^−/−^ deleted plus EgfrvIII viral overexpression) (Bruggeman et al., 2007). These cells stably express GFP from a constitutive promoter. *In vitro*, they express GSC markers, such as Nes, Sox2 and Olig2 (Fig. S3A). IENS cells generate aggressive infiltrative tumours when transplanted *in vivo* (*n*=4) (Fig. S3B). These were preferred to human patient-derived G7-GFP, as they displayed brighter GFP and were smaller, reducing needle blockage and therefore distributing better at the injection site. Moreover, use of mouse glioma cells is appealing for future studies, as this enables interrogation of certain immune responses (e.g. microglia activation).

IENS cells were injected precisely into the SEZ through the wall of the lateral ventricle. Initially, they displayed a remarkably specific localization and even distribution throughout the SEZ (Movie 1). We next compared cellular responses to the SEZ versus in other brain regions, scoring proliferation and astrocyte differentiation or quiescence (type B-like) (Ki67 and GFAP). Four distinct regions were tested: the cortex, CC, striatum and SEZ (Fig. 4B,C). As was the case with the human transplanted cells, we noted increased proliferation in the SEZ (Fig. 4B,C).

When imaged using confocal microscopy, we noted juxtaposition of GSCs with endothelial cells (Fig. 4E; Movie 2). The close interactions and extended processes along vessels is reminiscent of previous work reporting the importance of this as niche for NSCs (Kokovay et al., 2010). Five days after microinjection, the rate of proliferation was ∼40-50% (percentage of KI67 from the total GFP cells). We observed that ∼15% began to express high levels of the astrocytic marker GFAP (Fig. 4F). Cells remained viable for 2 weeks and showed evidence of proliferation and local infiltration into surrounding regions (*n*=3) (Fig. 4D; Movies 1 and 3).

### GSCs engrafted into brain slices respond to the cytostatic effects of TMZ

Whole brain slices harbouring GSCs provide a convenient method to explore the effects of pharmacological agents in an easier and higher throughput experimental system than live animals. To demonstrate proof of principle of its potential utility as a preclinical assay we first explored antimitotic treatments. Ara-C or TMZ have been widely used to assess NSC behaviour during regeneration and repair (Daynac et al., 2016; Doetsch et al., 1999). TMZ is the standard chemotherapy given to many patients with GBM. Both agents drive DNA damage and disrupt proliferation of tumour cells. TMZ is a DNA-alkylating agent that often induces G2/M arrest.

**Fig. 5.**
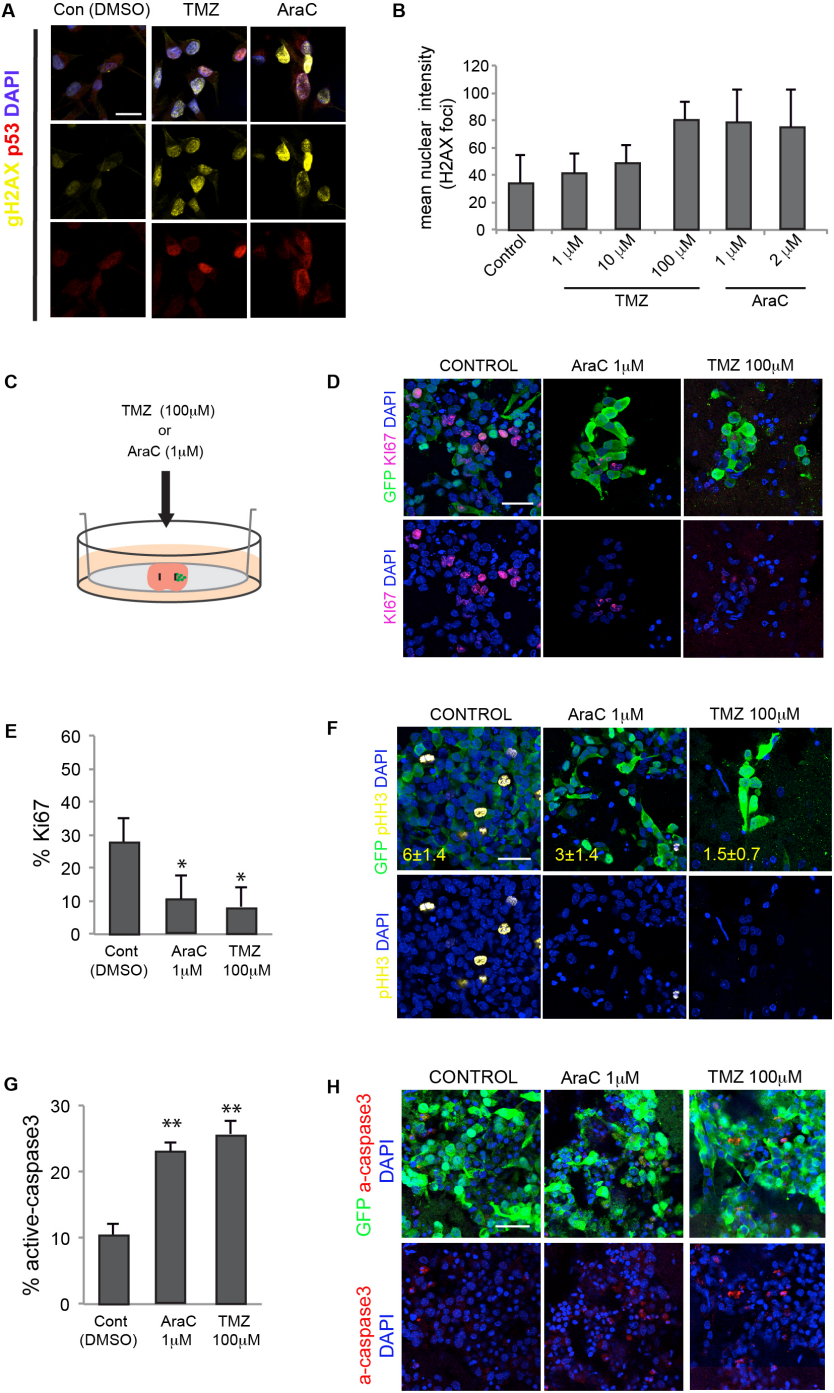
**TMZ and Ara-C treatment of slice co-cultures.** (A) DNA damage responses of mouse IENS-GFP cells following treatment with various does of the antimitotic agents AraC and TMZ. Immunocytochemistry for gamma-H2AX (gH2AX, yellow) and p53 (red). Nuclear counterstaining was performed with DAPI (blue) (B) Quantitation of the mean intensity of the nuclear H2AX foci (arbitrary units). (C) Schematic of the co-culture of IENS-GFP cells in the brain slices, adding pharmacological inhibitors of proliferation. (D) Immunostaining for GFP (green) and Ki67 (magenta) with DAPI (blue) for nuclear counterstaining. (E) Quantification of the percentage of Ki67^+^ (proliferative) cells using the proliferative inhibitors in IENS-GFP after 24 h. (F) Immunocytochemistry for GFP (green) and pHH3 (mitotic cells) (yellow) in IENS-GFP after treatments. Percentages of pHH3-positive cells are shown in yellow type. (G) Quantification of the percentage of active caspase 3 in the IENS-GFP cells after treatment with Ara-C and TMZ. (H) Immunostaining for active caspase 3 in the IENS-GFP cells in the brain slices after treatments. Scale bars: 20 µm in A and F; 30 µm in D and H. *n*=3, Student’s *t*-test, *P<0.05, **P<0.01.

Responses were scored using immunocytochemistry for two markers: gamma-H2AX for double-strand breaks and p53 as an indicator of DNA damage response (Fig. 5A). We first tested the activity of each factor at previously reported effective doses on IENS-GFP cultures *in vitro*, TMZ at 1, 10 and 100 µM, and the cytosine arabinoside (AraC) at 1 and 2 µM (Fig. 5A,B). We next treated slices harbouring successfully engrafted IENS-GFP cells after 3 days with 100 µM TMZ or 1 µM Ara-C (24 h) (Fig. 5C). Slices were then assessed for Ki67 and pHH3 using immunocytochemistry (Fig. 5D,E,F). In each condition, we observed a reduction in Ki67 and GFP double-positive cells (100 µM TMZ: 13%, 1 µM Ara-C: 20%). Untreated control slices remained ∼30% double positive (Fig. 5E,F). To determine the degree of cell death by apoptosis following treatment, we scored active caspase 3 (Fig. 5G,H). Cytostatic responses of tumour cells to drug treatment within slice co-cultures can therefore easily be monitored.

## DISCUSSION

Primary *in vitro* cell cultures of human GSCs are an important disease-relevant experimental model system. However, an obvious limitation of dissociated cell cultures is the difficulty in modelling interactions with the complex tissue environment. Here, we have demonstrated that brain tumour cell interactions with host brain tissue can be explored effectively by injection of cells onto adult brain slice cultures and co-culture.

Past studies have typically used microdissected regions of postnatal brain slices cultured in the presence of serum, as this is necessary to support long-term viability (months) (Ullrich et al., 2011). Yet our observations indicate that whole brain adult coronal slices are viable in serum-free medium for several weeks. This is long enough to permit tracking of tumour-host tissue interactions, such as interactions with endogenous stem cell niches or white matter tracts, and enables us to expose GSCs to brain tissue interactions. These methods therefore provide a tractable *ex vivo* model system that can now be exploited in both basic and translational studies of GBM. It is an approach that reduces the need for laborious and expensive mouse breeding or stereotaxic surgery, thereby increasing the speed and experimental throughput.

Avoiding exposure of transplanted cells to high levels of serum within traditional slice cultures methods enables a more physiological signalling environment to be maintained. This reduces the degree of serum-induced astrocyte differentiation which has hampered our previous studies (S.M.P., unpublished observations). Although serum-free medium has been used to maintain whole-mount tissue explants of the mouse SEZ for up to 16 h (Kokovay et al., 2010), to our knowledge, longer term survival of whole coronal brain slices in serum-free medium has not be reported or used in studies of GSCs. Surprisingly, we found that serum was not needed in order to maintain healthy slices of the whole adult coronal brain, at least for 1-2 weeks. Slices seem viable in the basal neural medium with no exogenous growth factors and supplemented only with N2 and B27 hormonal supplements.

With viable coronal adult brain slices, we were able to assess the responses of both mouse and human GSCs to distinct anatomical regions over several weeks of co-culture. Each mouse can provide up to five or six slices and cells can easily be injected in a spatially restricted manner. Live-cell imaging can be performed to track dynamic cell behaviours, such as interactions with blood vessels, infiltration or division (Movie 1). For example, GBM cells infiltrate widely, and have been shown to use both neuronal tracts and blood vessels as a substrate and guide for migration (Farin et al., 2006; Krusche et al., 2016). The slice cultures reported here should be useful in probing such distinct cellular mechanisms of infiltration. Another future potential application will be the tracking of cell lineage reporters, especially with the advent of genome editing tools that can be applied in GBM (Bressan et al., 2017). This will also help shed light on mechanisms of GSC dormancy and quiescence. One limitation of the method is that inevitably we trigger cell death and activation of the microglia through the slicing procedure itself; however, we did not notice region-specific differences across the slice, and most were located at the cell surface. As the slices are relatively thick (∼200 µm) and tumour cells infiltrate deep into the slice, it might be possible to explore tumour-cell microglia interactions.

We were particularly interested in assessing tumour cell responses within the SEZ. The SEZ provides a specific niche that sustains the NSCs, and a repertoire of cell-cell signals and ECM interactions that steer NSC fate, including endothelial cells, ependymal cells, and cerebral spinal fluid (produced by the choroid plexus) (Mirzadeh et al., 2008; Shen et al., 2008; Silva-Vargas et al., 2016). Gliomas might frequently arise from endogenous neural stem cells, or serve as a reservoir of cells that in some patients can drive relapse (Piccirillo et al., 2015). We noted key elements of the healthy SEZ microenvironment that were viable: the ventricle, gliotubes, rostral migratory stream (RMS) and ependymal cells. Importantly, endothelial cells within this region are thought to serve as an important niche signal; indeed, we noted close interactions between vessels and tumour cells, with extended processes and wrapping around the vessel, highly reminiscent of normal NSC interactions (Kokovay et al., 2010).

We demonstrated that human GSCs can engraft effectively into the tissue of SEZ. CD133 is expressed by many GSCs. We observed expression of this marker in both the mouse and human GSCs. Another more recently proposed marker of the quiescent astrocytes is CD9. We and others have recently found increased levels of CD9 in GSCs compared to normal NSCs (Okawa et al., 2016; Podergajs et al., 2015). We found that CD9 is indeed retained on cells within the SEZ, suggesting that ‘stemness’ can be effectively retained. As anticipated, reduced proliferative responses were noted when cells were deposited at other anatomical sites, such as the CC. Distinct brain regions clearly will have significant differences in their ability to influence tumour cell proliferation and differentiation.

A limitation of this slice culture approach is the difficulty of achieving viable slice cultures past 3 weeks. Although we did note some loss of some tissue integrity past 3 weeks, we did not specifically push this further or search for modified culture regimes. This might be important to resolve in future, particularly for slower growing human GSCs, which take weeks to months to initiate tumour growth in live xenografted mice. Alongside the damage and immune activation triggered by the slice procedure itself and inherent disadvantages, it means that this assay cannot replace either GEMMs or intracranial transplantation in live mice.

A multitude of new agents are emerging that will require effective preclinical studies. There is a bottleneck and huge cost associated with the testing of new pharmacological agents in living animals before they can enter clinical trials for GBM. The methods outlined here offer a potential complementary assay to *in vivo* testing. To demonstrate potential utility as a preclinical model, we tested the responses of cells to antimitotics (AraC and TMZ). TMZ is used in many GBM treatment strategies, yet our understanding of how it influences distinct compartments of the GSC and resistance mechanisms remains limited. Future candidate drugs will need to be explored alongside TMZ to search for the most effective doses and regimes. Thus, the methods reported here should therefore prove useful in the prioritising and triage of candidate therapeutic agents moving through preclinical studies.

In conclusion, the organotypic method presented here provides a simplified model for assessing responses of GSCs to various brain anatomical sites and microenvironments. This will therefore complement existing *in vitro* and *in vivo* models, helping to prioritize genes and pathways controlling key malignant properties of GBM and aiding the preclinical testing of new anticancer agents.

## MATERIALS AND METHODS

### Organotypic adult brain slice culture

C57BL/6 mice (aged 5-8 weeks) were used. More consistent results were often obtained using the younger animals, particularly in viability after 2-3 weeks’ culture. The brain was removed from the skull and transferred to a 10-cm^2^ tissue culture dish with sterile PBS and placed on ice (Fig. 1A,B). The cerebellum and olfactory bulb were removed (Fig. 1B), and remaining forebrain was transferred into a 35-mm^2^ dish with pre-warmed 3% SeaPlaque™ agarose (50100, Lonza) (Fig. 1A,B). Upon cooling in ice, the block was removed and cut using a scalpel into a ∼2 cm cube around the brain. Before starting to cut, a six-well plate was prepared. In each well, we introduced one cell culture insert (PICMORG50, Millicell) and added below it 1 ml culture medium in serum-free basal NSC medium, Dulbecco's modified Eagle medium:F12 supplemented with N2 and B27 (Life Technologies). The embedded brain was placed in the circular vibratome plate with glue (Fig. 1A,B). The vibratome (VT1000 S, Leica) plate was fixed in the platform and filled with PBS with penicillin-streptomycin (15140-122, Gibco, 1:100). Then, 250 µm thick slices were cut, with vibrating frequency set at 8 and speed at 3. Each slice was transferred using a small brush onto the top of a Millipore culture insert (Fig. 1A,B). Six slices were cut per animal along the SEZ. The platform was maintained cooled at all times. We obtained six slices around the forebrain ventricle. The six-well plate was placed in an incubator at 37°C+5% CO_2_, and slices were incubated for 24-48 h prior to cell transplantation.

### Glioblastoma cancer stem cell transplantation onto brain slices

G7 human GBM stem cell cultures have been previously described (Stricker et al., 2013). For human cell transplants, a standard Gilson pipette was used to deposit 0.2 µl of the cells onto the centre of the striatum, the typical injection site when performing stereotaxic surgery for tumour initiation assays. These cells engrafted well into the slice and their integration could be observed the following day.

IENS-GFP mouse cells were a gift from Dr Lohuizen (Bruggeman et al., 2007). Both mouse and human GNS cells were grown using conditions previously described (Pollard et al., 2009). After centrifugation, cells were harvested and resuspended in medium at 100,000 cells/µl. Cells were used immediately for transplantation in the SEZ. Two different methods of cell injection were used. A manual using a p2 Gilson pipette was used to inject 0.5 µl, while an auto-nanoliter injector (Nanoject II, Drummond Scientific Company) was used for 40-100 nl injections. For the injector, we used glass capillaries pulled using an automated needle puller (tip diameter, 10-20 μm; Drummond) (Fig. 1B); 4000 cells were transplanted per injection, and 20,000 cells were transplanted when the P2 pipette was used. To facilitate engraftment and prevent wide dispersion of the 0.2 µl of cells, we used forceps to make a small indentation in the transplant site prior to delivery of the cells. Slices with cells were incubated at 37°C+5% CO_2_ for 7 days, with fresh NSC medium (no EGF or FGF-2) added every 2 days. Cell engraftment of the cells was observed 4 days after transplantation and cells were monitored using a fluorescence stereo microscope (M165 FC, Leica). For antimitotic treatments in the IENS-GFP *in vitro*, different doses of AraC (1 µM, 2 µM; Sigma-Aldrich) and TMZ (1 µM, 10 µM and 100 µM; Sigma-Aldrich) were added to the complete medium for 24 h. For antimitotic treatments of IENS-GFP in the slices, the cells were growing in the brain slice for 3 days, and AraC (1 µM) and TMZ (100 µM) were added to the complete medium for 24 h.

### Immunocytochemistry

Medium was removed and exchanged for 1 ml of freshly prepared 4% paraformaldehyde (PFA), and 1-2 ml PFA was also placed gently on top to cover the slice. After 2 h, PFA was removed and brain slices were washed with PBS three times. Slices were transferred using a brush to a 24-well plate. Slices were incubated at room temperature for 1.5 h in blocking solution (0.2% Triton X-100 and 3% goat serum; Sigma-Aldrich). Primary antibodies were as follows: anti-GFAP (G3893, Sigma-Aldrich, 1:100), anti-Ki67 (RM-9106, Thermo Fisher Scientific, 1:100), anti-GFP (13970, Abcam, 1:100), anti-CD31 (14-0311-81, eBiosience, 1:100), anti-CD9 (14-0091-82, eBioscience, 1:500), anti-CD133 (MAB4310, Millipore, 1:100), anti-DCX (AB2253, Millipore, 1:500), anti-nestin (Rat 401, Developmental Studies Hybridoma Bank, 1:10), anti-stem121 (440410, Cellalartis, 1:300), anti-pH3 (50-9124-41, eBioscience, 1:50), anti-H2AX (phosphor S139) (ab81299, Abcam, 1:50), anti-Iba1 (NB-1-1028SS, Novus Biologicals, 1:100), anti-Olig2 (AB9610, Millipore, 1:200), anti-Sox2 (AB5603, Millipore, 1:100), anti-active caspase 3 (ab2302, Abcam, 1:100).

For H2AX staining, cells were fixed with methanol-acetone 1:1 for 10 min at room temperature. Positive cells were scored using Fiji image analysis software. For immunohistochemistry, the primary antibodies were incubated for 2 days at 4°C. After three washes with PBS, slices were incubated with appropriate Alexa Fluor-conjugated (Life Technologies) secondary antibody and DAPI (D9542, Sigma-Aldrich) for 4-6 h. Slices were washed three times, mounted on slides, and immersed in FluoroSave™ Reagent (345789, Calbiochem). Slices were examined with a confocal microscope (TCS SP8, Leica). PI (14289-25, Cayman Chemical) was used at 5 µg/ml in PBS for 5 min and the tissue was analysed under a fluorescent stereomicroscope. For Haematoxylin and Eosin (H&E) staining, brain slices were fixed with 4% PFA for 1 h. They were immersed in Harris Haematoxylin (6765004, Thermo Fisher Scientific) for 10 min and then washed in tap water. Following this, the slices were immersed for 10 s in 1% acid-alcohol and washed again in tap water. The brain slices were washed in tap water for 30 s and washed again in tap water. Finally, they were immersed in Eosin Y (6766010, Thermo Fisher Scientific) for 10 s and washed once more. The brain slices were imaged using a Leica stereomicroscope.

## Supplementary Material

Supplementary information

## Supplementary Material

First Person interview
