# Supplementary material for: Modelling glioblastoma tumour-host cell interactions using adult brain organotypic slice co-culture
Source: Dis Model Mech. 2018 Feb 1;11(2):dmm031435. doi: 10.1242/dmm.031435 (PMC5894940; doi:10.1242/dmm.031435)
Supplement: Supplementary information [file dmm-11-031435-s1.pdf]

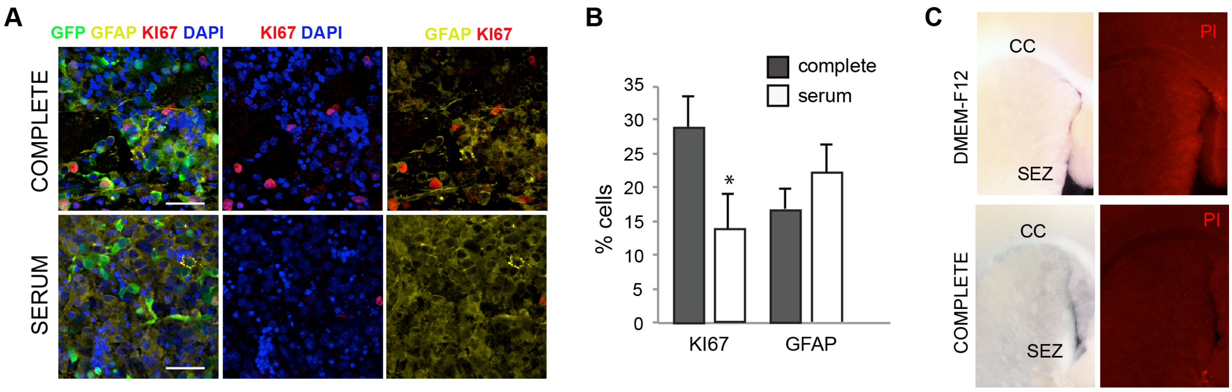

Supplementary Figure 1. (A) Immunostaining for GFAP (red) and Ki67 (magenta) of IENS-GFP cells deposited in the brain slices with complete media with/without serum. (B) Quantification of the percentage of cells positive for GFAP and KI67. (C) PI staining in brain slices after 24 hours. Slices were cultured with DMEM-F12 or with complete media. Scale bar in A: 30 µm. N=3, Students t-test p<0.01.

Disease Models & Mechanisms • Supplementary information

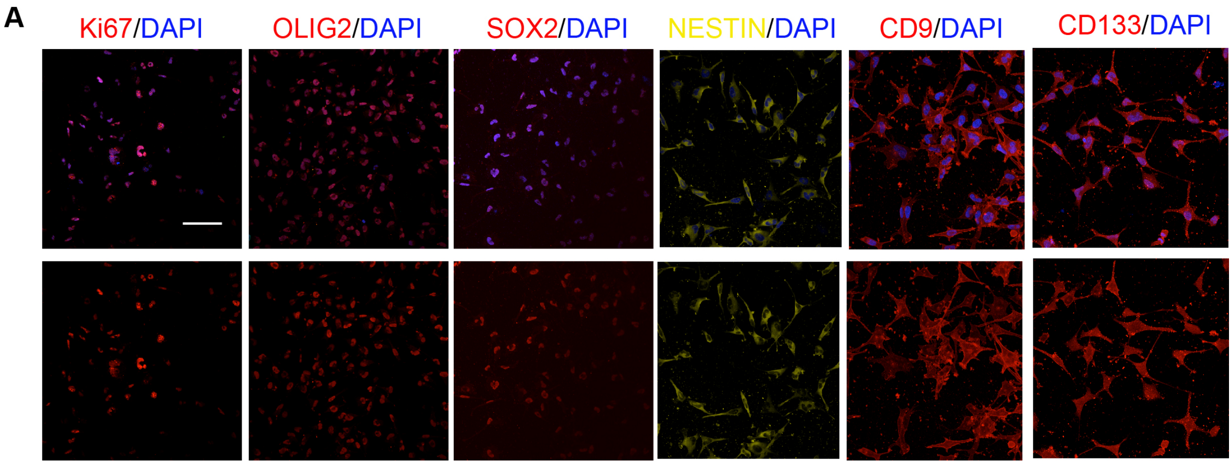

Supplementary Figure 2. (A) G7cells in vitro. Immunostaining for different markers in vitro. KI67 (red), Olig2 (red), Sox2 (red), Nestin (yellow), CD9 (red), CD133 (red). Scale bar: A= 20 µm

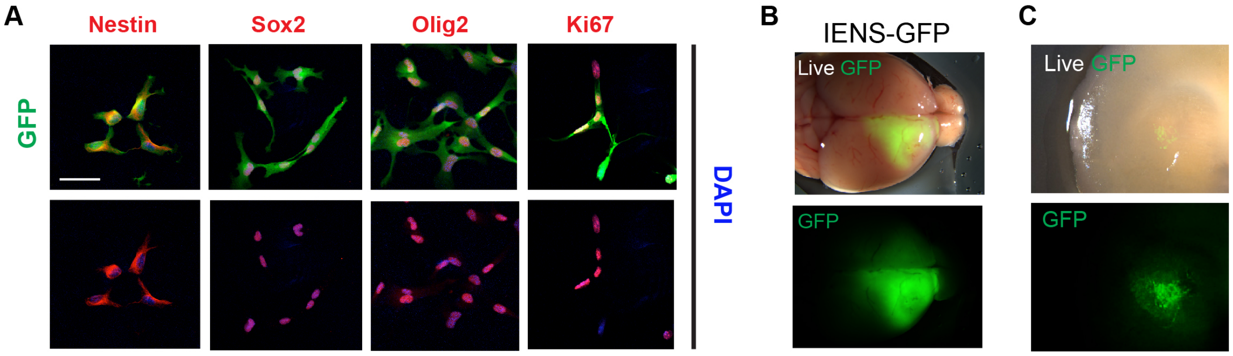

Supplementary Figure 3. IENS-GFP cells on the brain tissue in complete media or complete media supplemented with serum after 5 days (A) Immunostaining of GFAP (red) and KI67 (magenta) in IENS-GFP. (B) Quantification of the percentage of KI67 and GFAP positive cells. Scale bar in A: 20 μm.

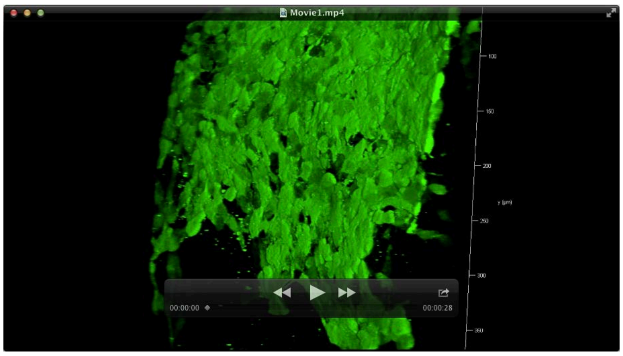

Movie 1 Figure 2. Live IENS-GFP cells infiltrating through the brain tissue after 10 days.

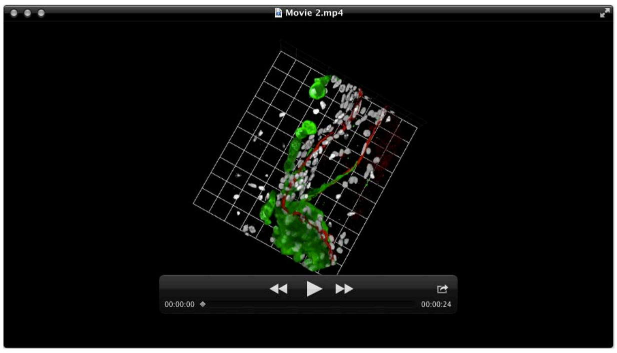

Movie 2 Figure 2. 3D reconstitution of the IENS-GFP cells (green) engraftment in the SEZ after 5 days.

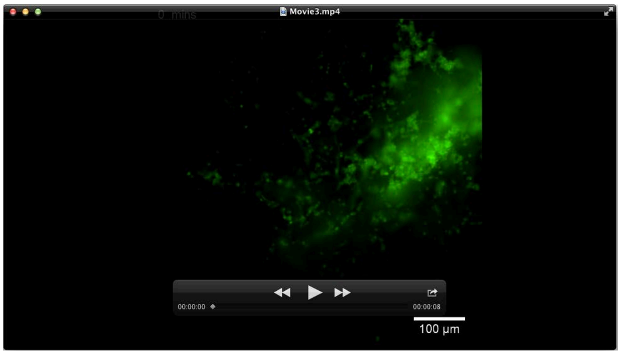

Movie 3 Figure 2. 3D reconstruction of close interaction between the IENS-GFP cells (green) and endothelial cells (red). Nuclear counterstaining with DAPI (blue or grey).
