# Supplementary material for: Modelling glioblastoma tumour-host cell interactions using adult brain organotypic slice co-culture
Source: Dis Model Mech. 2018 Feb 1;11(2):dmm031435. doi: 10.1242/dmm.031435 (PMC5894940; doi:10.1242/dmm.031435)
Supplement: First Person interview [file dmm-11-031435-s2.pdf]

© 2018. Published by The Company of Biologists Ltd | Disease Models & Mechanisms (2018): doi:10.1242/dmm.031435: Supplementary information

FIRST PERSON

First person – Maria Angeles Marques-Torrejon

First Person is a series of interviews with the first authors of a selection of papers published in Disease Models & Mechanisms, helping early-career researchers promote themselves alongside their papers. Maria Angeles Marques-Torrejon is first author on 'Modelling glioblastoma tumour-host cell interactions using adult brain organotypic slice co-culture', published in DMM. Maria is a Postdoctoral Researcher in the lab of Steven Pollard at the MRC Centre for Regenerative Medicine and Edinburgh Cancer Research UK Cancer Centre, University of Edinburgh, UK, investigating relapse in glioblastoma.

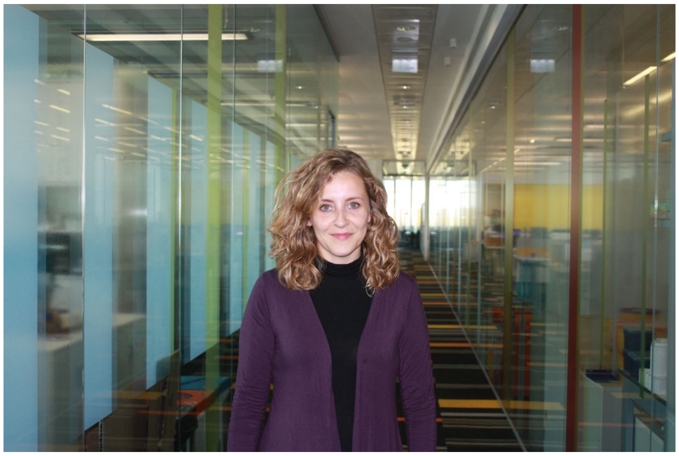

Maria Angeles Marques-Torrejon

How would you explain the main findings of your paper to non-scientific family and friends?

Glioblastoma (GBM) is an aggressive incurable brain cancer. We wanted to generate a system for easier and cheaper analysis of cells that fuel the tumour. They are called glioma stem cells (GSCs). With our system, we are able to track and analyse these cells on adult mouse brain slices. We could analyse how they proliferate and interact with blood vessels and how they are able to respond to the anti-mitotic drug temozolomide as proof-of-principle of the utility in modelling responses to existing treatments.

What are the potential implications of these results for your field of research?

Our goal was to design a system that allows us to understand the mechanisms that regulate relapse in GBM. There is a population of GSCs that are in quiescence state and are resistant to cytotoxic therapies. After a time they are able to regenerate the tumour. We know that some GSCs are expressing quiescence markers in our system. Then, we will be able to use anti-mitotic drugs and to know more about which mechanisms regulate the relapse in GBM.

"It's fundamental to have motivational people around you, as science can be really frustrating."

What are the main advantages and drawbacks of the model system you have used as it relates to the disease you are investigating?

The advantages of our system are that we are able to analyse single cells and the different intermediate times in tumour formation. It is also cheaper and faster that transplants in mice. The drawbacks of the model system are the limited timing for analysis due to the viability of the tissue and maybe the possibility of contamination, but for this we worked carefully using antibiotics during the generation of the brain slices.

What has surprised you the most while conducting your research?

The maintenance of brain tissue viability for weeks and all the possibilities of study that the system offers with the human and mouse GBM lines.

Describe what you think is the most significant challenge impacting your research at this time and how will this be addressed over the next 10 years?

Maybe the most significant challenge in this research would be to use this system in personalized clinical assays. Then, this system could be used for screening of pharmacological inhibitors of proliferation. For example, after biopsy of GBM patients, their cells could be cultured in our system to know which anti-mitotic drug is working better in order to improve the treatment.

What changes do you think could improve the professional lives of early-career scientists?

I think that at the beginning of a scientist's career it is most important to have an accessible and motivated supervisor during

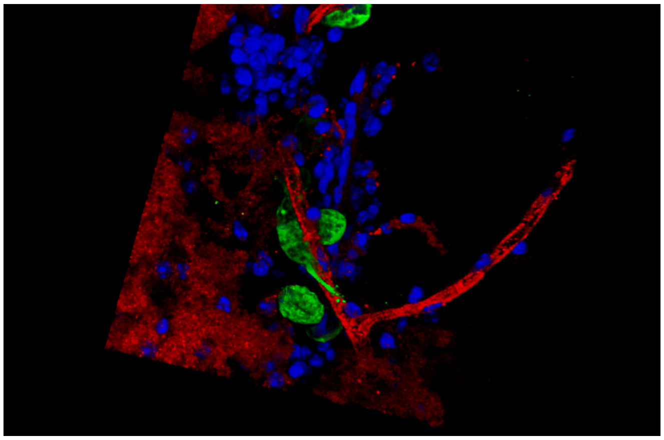

Glioblastoma cells interacting with a blood vessel.

Maria Angeles Marques-Torrejon's contact details: MRC Centre for Regenerative Medicine and Edinburgh Cancer Research UK Cancer Centre, University of Edinburgh, UK.
E-mail: Maria.Marques@ed.ac.uk

your PhD. Another point is a clearly defined framework for a principal project; there will be enough time afterwards for side projects. It's fundamental to have motivational people around you, as science can be really frustrating. On the other hand, the scientific career, as others, depends on the time to reach certain goals. In the case of women, it is very hard to reconcile the role of mother with that of scientist, especially when you are a postdoc and you are supposed to give 100% to production. I think that we should be given certain facilities to make everything easier.

What's next for you?

My immediate scientific goals are to further develop this model in the relapse of GBM context. Professionally, I am looking forward to finishing the principal project of my postdoc before maternity leave.

Reference

Marques-Torrejon, M. A., Gangoso, E. and Pollard, S. M. (2018). Modelling glioblastoma tumour-host cell interactions using adult brain organotypic slice co-culture. *Dis. Model. Mech.* **11**, doi:10.1242/dmm.031435.
